# Macrophage Resistance to Ionizing Radiation Exposure Is Accompanied by Decreased Cathepsin D and Increased Transferrin Receptor 1 Expression

**DOI:** 10.3390/cancers15010270

**Published:** 2022-12-30

**Authors:** Ana Teresa Pinto, Ana Beatriz Machado, Hugo Osório, Marta Laranjeiro Pinto, Rui Vitorino, Gonçalo Justino, Cátia Santa, Flávia Castro, Tânia Cruz, Carla Rodrigues, Jorge Lima, José Luís R. Sousa, Ana Patrícia Cardoso, Rita Figueira, Armanda Monteiro, Margarida Marques, Bruno Manadas, Jarne Pauwels, Kris Gevaert, Marc Mareel, Sónia Rocha, Tiago Duarte, Maria José Oliveira

**Affiliations:** 1 i3S–Instituto de Investigação e Inovação em Saúde, Universidade do Porto, 4200-135 Porto, Portugal; 2 INEB–Instituto de Engenharia Biomédica, Universidade do Porto, 4200-135 Porto, Portugal; 3 Department of Medical Sciences, Institute of Biomedicine (iBiMED), Universidade de Aveiro, 3810-193 Aveiro, Portugal; 4 Champalimaud Centre for the Unknown, Fundação Champalimaud, 1400-038 Lisboa, Portugal; 5 IPATIMUP–Instituto de Patologia e Imunologia Molecular da Universidade do Porto, 4200-135 Porto, Portugal; 6 Departament of Pathology, Faculdade de Medicina, Universidade do Porto, 4200-319 Porto, Portugal; 7 Centro de Química Estrutural–Institute of Molecular Sciences, Instituto Superior Técnico, Universidade Técnica de Lisboa, 1049-001 Lisboa, Portugal; 8 CNC–Center for Neuroscience and Cell Biology, Universidade de Coimbra, 3004-504 Coimbra, Portugal; 9 Institute for Interdisciplinary Research (III), Universidade de Coimbra, 3030-789 Coimbra, Portugal; 10 REQUIMTE–LAQV, Chemistry Department, NOVA School of Science and Technology, Universidade de Lisboa, 2829-516 Caparica, Portugal; 11 Personal Health Data Science Group, Sano-Centre for Computational Personalised Medicine, 30-054 Krakow, Poland; 12 Radiotherapy Service, Centro Hospitalar Universitário São João (CHUSJ), EPE, 4200-319 Porto, Portugal; 13 VIB-UGent Center for Medical Biotechnology, 9052 Ghent, Belgium; 14 Department of Biomolecular Medicine, Ghent University, 9052 Ghent, Belgium; 15 Department of Radiation Oncology and Experimental Cancer Research, Ghent University Hospital, 9000 Ghent, Belgium; 16 Institute of System, Molecular and Integrative Biology, University of Liverpool, Liverpool L69 3 GE, UK; 17 IBMC–Instituto de Biologia Molecular e Celular, Universidade do Porto, 4200-135 Porto, Portugal

**Keywords:** macrophages, ionizing radiation, radiotherapy, proteomics, cathepsin D, transferrin receptor 1 (TfR1), iron metabolism

## Abstract

**Simple Summary:**

Resistance to treatment, particularly to radiotherapy, is still a major clinical problem in cancer management. Macrophages are abundant immune cells at the tumor microenvironment, being exposed to ionizing radiation during cancer radiotherapy. Considering the role of macrophages in tumor progression and therapy outcome, it is crucial to investigate their response to clinically relevant ionizing radiation doses for the design of new strategies to overcome tumor radio resistance. In this work, we have used a proteomic approach to evaluate the expression profile of irradiated versus non-irradiated macrophages. This analysis, supported by validation using cell-based assays, led to the identification of two main deregulated targets, cathepsin D and transferrin receptor 1, in irradiated macrophages. Investigating macrophage response to ionizing radiation could lead to the identification of deregulated pathways and molecular players that can be targeted to overcome tumor radio resistance.

**Abstract:**

Purpose: To identify a molecular signature of macrophages exposed to clinically relevant ionizing radiation (IR) doses, mirroring radiotherapy sessions. Methods: Human monocyte-derived macrophages were exposed to 2 Gy/ fraction/ day for 5 days, mimicking one week of cancer patient’s radiotherapy. Protein expression profile by proteomics was performed. Results: A gene ontology analysis revealed that radiation-induced protein changes are associated with metabolic alterations, which were further supported by a reduction of both cellular ATP levels and glucose uptake. Most of the radiation-induced deregulated targets exhibited a decreased expression, as was the case of cathepsin D, a lysosomal protease associated with cell death, which was validated by Western blot. We also found that irradiated macrophages exhibited an increased expression of the transferrin receptor 1 (TfR1), which is responsible for the uptake of transferrin-bound iron. TfR1 upregulation was also found in tumor-associated mouse macrophages upon tumor irradiation. In vitro irradiated macrophages also presented a trend for increased divalent metal transporter 1 (DMT1), which transports iron from the endosome to the cytosol, and a significant increase in iron release. Conclusions: Irradiated macrophages present lower ATP levels and glucose uptake, and exhibit decreased cathepsin D expression, while increasing TfR1 expression and altering iron metabolism.

## 1. Introduction

Although radiotherapy is a widely used anticancer treatment modality, treatment resistance is still a major challenge [1]. The key to improve radiotherapy efficacy may rely on fundamental biology, mainly on a better understanding of the effect of ionizing radiation (IR) on non-cancer cells that constitute the tumor microenvironment. These are comprised within the irradiated region and are therefore exposed to the same IR dose as cancer cells during radiotherapy sessions.

Macrophages are particularly abundant cells at the tumor microenvironment, constituting the major inflammatory stromal component in many tumors [2]. Macrophages have been described as obligate partners for cancer cell migration, invasion, and metastasis [2], being also involved in the response to chemotherapeutic and immunotherapeutic agents, which makes them excellent targets to improve anti-cancer therapies [3,4,5]. During radiotherapy, IR induces the production of death signals by cancer cells, leading to the recruitment of more immune cells, including monocytes [6]. At the injured site, monocytes differentiate into macrophages helping to clear dying cells after tissue irradiation [7]. Exposure to therapeutic IR doses promotes macrophage activation towards a pro-inflammatory phenotype, which contributes to anti-tumor immune response through different molecular mechanisms [8]. However, macrophages seem to display a radiation-resistant phenotype and may also contribute to tumor resistance to radiotherapy [9,10], which highlights the need to better understand macrophage biological response to IR.

Most studies addressing the macrophage response to IR have used distinct macrophage models (mainly of non-human origin) [11,12,13], different types of radiation [14,15] and a wide range of single doses [16,17,18,19], from low (<0.1 Gy) [20] to moderate (0.1 Gy–1 Gy). Although some specific radiotherapy schemes involve the delivery of high singles doses (usually >10 Gy), most patients are usually exposed to a multi-fractionated regimen (5×/week), with daily doses of typically 2 Gy, reaching a cumulative dose of 50–70 Gy (conventional), or larger fractions given over a shorter period of time (hipofractionated schemes) [21,22]. Thus, the experimental models that most likely resemble the multi-fractionated regimen are very useful for assessing the clinical effect of IR on macrophages. We previously established an experimental model system that mimics a week of a cancer patient’s treatment, by exposing primary human monocyte-derived macrophages to cumulative X-ray fractions (2 Gy/fraction/day) for 5 days. Thereby, we demonstrated that irradiated macrophages remained viable and metabolically active, still promoting cancer cell invasion and cancer cell-induced angiogenesis, which are major concerns that need to be addressed to improve radiotherapy efficacy [23]. We also demonstrated that, depending on the cancer cell line macrophages are co-cultured with, they could either promote or decrease cancer cell apoptosis after radiation exposure, and differently regulate the expression of pro- or anti-inflammatory markers [24]. However, the main molecular mechanisms involved in macrophage response to radiation have not yet been identified. 

Although proteomics has gained a special interest in the field of radiation biology [25], only a few studies investigated the macrophage response to IR exposure using mass spectrometry-based proteomics. In 1999, Stulík and colleagues studied the effects of low-dose IR in a monocytic-derived cell line, identifying global radiation-induced phosphorylation and expression protein changes [26]. In 2005, Chen et al. demonstrated increased expression of actin cytoplasmic 1 in vivo, upon mouse exposure to a single whole-body dose of 0.5 Gy [27]. In 2009, Smallwood and colleagues identified an IR dose-dependent increase in the expression of the calcium regulatory protein calmodulin (CaM) in mouse macrophages (RAW 264.7) [28]. CaM overexpression was suggested to increase DNA repair pathways, enhancing macrophage radio resistance. Although these studies provide important data, it is difficult to speculate whether a similar response would be observed in human macrophages, as mouse and human macrophages present many distinct features [29].

In the present work, we used our previously established in vitro model of human monocyte-derived macrophages exposed to the clinically relevant IR scheme of 5 × 2 Gy [23] and assessed protein expression changes by contemporary mass spectrometry-driven proteomics. The identified targets are associated with cell metabolism and regulation of localization. We validated the statistically significant downregulation of cathepsin D and the upregulation of the transferrin receptor 1 (TfR1) expression in irradiated macrophages.

The obtained knowledge appoints novel molecular targets potentially involved in macrophage response to radiation, whose modulation may enhance cancer cell radiation sensitivity, thereby increasing radiotherapy efficacy.

## 2. Materials and Methods

### 2.1. Macrophage Culture and Exposure to IR 

Macrophages were differentiated from human blood monocytes isolated from buffy coats and exposed to 50 ng/mL of macrophage colony-stimulating factor (M-CSF) (ImmunoTools), as previously described [23]. Macrophages, from the same donor, were exposed (IR) or not (Ctr) to X-ray doses (2 Gy/fraction/day), for 5 days (5 × 2 Gy), totalizing a 10 Gy cumulative dose. Macrophage primary cultures obtained from different blood donors were considered biological replicates. Macrophage irradiation experiments were performed in collaboration with the Radiotherapy Service of CHUSJ, as previously described [23]. Briefly, using a radiotherapy treatment planning system (ELEKTA CMS XiO v.4.7.0), a dosimetric plan was established to deliver the desired dose with photon beams produced in a clinical linear particle accelerator (Siemens PRIMUS), operated at 6 or 18 MV.

### 2.2. Cell Metabolic Activity

Macrophage metabolic activity was determined through resazurin reduction assay. Briefly, 20 h after radiation exposure (5 × 2 Gy), macrophages were incubated with the resazurin redox dye (0.01 mg/mL) (Sigma-Aldrich, St. Louis, MO, USA) for 3 h, at 37 °C and 5% CO_2_. After resazurin reduction, its fluorescence was measured (530 nm Ex/590 nm Em) using the multi-mode microplate reader Synergy MX (BioTek, Winooski, VT, USA).

### 2.3. Glucose Uptake 

Glucose levels were measured in the conditioned medium (CM) from irradiated (5 × 2 Gy) and non-irradiated macrophages (*n* = 12), collected 24 h after irradiation. Briefly, CM was incubated with reagent 1 (mti-Diagnostics), composed of phosphate buffer, phenol, glucose oxidase, peroxidase and 4-amino-antipyrine, for 20 min at room temperature (RT). In the first step, glucose was converted into D-glucono-1,5-lactone plus hydrogen peroxide by glucose oxidase, used for the second step, when peroxidase generated a colored product, whose intensity is proportional to the sample glucose concentration. The absorbance was read at 500 nm with the multi-mode microplate reader Synergy MX (BioTek, Winooski, VT, USA). Glucose concentration values were subtracted from that of the RPMI 1640 medium, which is equivalent to 11.11 nM, to estimate glucose uptake. Data from irradiated macrophages were compared to those of non-irradiated ones and expressed as fold-change.

### 2.4. Lactate Levels

Briefly, 24 h after irradiation, CM from irradiated (5 × 2 Gy) macrophages and their non-irradiated counterparts (*n* = 14) was collected and incubated with working reagent (Spinreact) for 10 min at RT. Lactate was then oxidized by lactate oxidase to pyruvate and hydrogen peroxide, which in the presence of peroxidase and the remaining reagent compounds forms a red quinone product. The intensity of the color formed is proportional to the lactate concentration in the sample. The absorbance was read at 505 nm with the multi-mode microplate reader Synergy MX (BioTek, Winooski, VT, USA). A lactate standard (1.123 nmol/L) was used as a reference value. Finally, data were normalized to CM protein concentration, and lactate levels of irradiated macrophages were then compared to those of non-irradiated ones and expressed as fold-change.

### 2.5. Total Levels of Cellular ATP 

Total levels of cellular ATP were measured with the Luminescent ATP Detection Assay Kit (MitoSciences-Abcam, Cambridge, UK) according to the manufacturer´s instructions. Briefly, 24 h after the last irradiation dose, irradiated macrophages (5 × 2 Gy) (*n* = 6 donors) and their non-irradiated counterparts (*n* = 6) were lysed with detergent, allowing ATPases to be irreversibly inactivated, and incubated with a substrate solution that reacts with ATP. The emitted light was proportional to the ATP concentration inside the cell. Luminescence was then measured with the multi-mode microplate reader Synergy MX (BioTek, Winooski, VT, USA). ATP values of each sample were obtained from a standard curve previously performed with ATP dilution series and normalized to CM protein concentration. 

### 2.6. Iron Supplementation 

Irradiated and non-irradiated macrophages were incubated with 100 μM of ferric ammonium citrate (FAC) (Sigma-Aldrich, St. Louis, MO, USA) in fresh complete cell culture medium for 24 h, after the last irradiation. For determination of intracellular iron content and protein expression analysis, after supplementation, macrophages (*n* = 6 donors) were washed twice with PBS supplemented with 20 μM desferrioxamine (DFO), an extracellular iron chelator, and lysed. For determination of iron release, iron-supplemented macrophages (*n* = 12 donors) were incubated in serum-free medium with 50 μM DFO for additional 2 h, which prevented iron re-uptake, after which the CM was collected.

### 2.7. Iron Quantification

To measure intracellular iron, macrophages were supplement with iron, as described above, after which they were detached using accutase (PAA Laboratories) and counted using a TC20™ Automated Cell Counter (BioRad, Hercules, CA, USA). Macrophages were then lysed using 65% HNO_3_ for 1.5 h, followed by the addition of 30% H_2_O_2_ for additional 3.5 h, at 80 °C. Cell lysates were then diluted with deionized water to obtain a final concentration of 5% HNO_3_. Iron release was directly determined in the collected CM. Iron levels were quantified using Inductively Coupled Plasma–Atomic Emission Spectroscopy (ICP-AES) (Ultima model, Jobin Yvon HORIBA) at 259.940 nm. Calibration was performed using Fe standards from 0.1 to 1 mg/L, diluted in the same matrix as used for the analyzed samples, i.e., complete cell medium supplemented with DFO, for CM, and 5% HNO_3_, for cell lysates. Iron levels were normalized to the number of cells.

### 2.8. Proteomics

Proteins from irradiated and non-irradiated macrophages, obtained from the same blood donor, were extracted using lysis buffer [50 mM Tris-HCl (pH 7.5), 150 mM NaCl, 2 mM EDTA and 1% Igepal], supplemented with a cocktail of proteases and phosphatases inhibitors: phenylmethanesulfonylfluoride 1 mM, sodium metavanadate 3 mM, sodium fluoride 20 mM, sodium pyrophosphate tetrabasic 25 mM (AppliChem, Darmstadt, Germany), aprotinin 10 mg/mL and leupeptin 10 mg/mL (Sigma-Aldrich, St. Louis, MO, USA). The protein concentration was determined with Protein Assay Dye Reagent Concentrate (Bio-Rad, Hercules, CA, USA). Protein lysates were performed 24 h after IR (5 × 2 Gy) exposure.

About 1 mg of protein was precipitated with acetone (1:8) (*v*/*v*) and kept at −80 °C for 15–20 min. Precipitated proteins were then centrifuged at 20,000× *g* for 15 min and re-suspended in 0.5 M of triethylammonium bicarbonate buffer (TEAB) (Sigma-Aldrich, St. Louis, MO, USA) pH 8.5, and vortexed. To better dissolve the pellet, samples were sonicated for 2 min in a cup horn at 20% and then at 40% amplitude, 1 s ON and 1 s OFF cycle (Vibra Cell 750 watt, Sonics & Materials, Newtown, CT, USA). The protein content was quantified using the 2-D Quant Kit (GE Healthcare, Chicago, IL, USA), according to manufacturer´s instructions. Protein (100 µg) was concentrated in a rotary evaporator (Concentrator Plus, Eppendorf, Hamburgo, Germany) at 60 °C. Protein pellets were dissolved in 0.5 M TEAB to a final volume of 90 µL, and 8 µL of 50 mM tris (2-carboxyethyl) phosphine hydrochloride (TCEP) (Sigma-Aldrich, St. Louis, MO, USA) was added and sonicated in a cup horn for 1 min at 20% amplitude to facilitate protein denaturation. Then, 4 µL of 200 mM methyl methanethiosulfonate (MMTS) (Sigma-Aldrich, St. Louis, MO, USA) was added, and samples were incubated for 10 min at RT. Further, 0.5 M TEAB was added to a final volume of 190 µL and the sample was vortexed. Protein digestion was performed by adding 10 µL of trypsin (Roche) (0.5 µg/µL), diluted in 0.5 M TEAB, to reach a 1:20 (w:w) enzyme:protein ratio, followed by overnight incubation at 37 °C. After digestion, 2 µL of formic acid was added and samples were then dried by rotary evaporation under vacuum for 1 h at 60 °C. The samples were solubilized in 75 µL of 70% isopropanol/30% TEAB and sonicated for 10 min at 20% amplitude, with pulses of 1 s ON and 1 s OFF. Digested peptides were then labeled with the iTRAQ (8-plex) tags according to the manufacturer´s instructions (Applied Biosystems, Waltham, MA, USA). Eight samples from 4 blood donors were used in the 8-plex analysis: 4 samples from irradiated (5 × 2 Gy) macrophages (labeled with 114, 116, 118, 121 reporters) and another 4 corresponding to non-irradiated macrophages (labeled with 113, 115, 117 and 119 reporters), which were then combined into a single mixture.

About 650 µg of each peptide sample was solubilized in 2% acetonitrile (ACN) in 72 mM TEAB and fractionated by high pH reverse phase chromatography using an UltimateTM3000 LC (LC Packings, Dionex, Sunnyvale, CA, USA) with two online Aeris 3.6 µm XB-C18 columns (15 cm × 2.10 mm, Phenomenex, Torrance, CA, USA), using 72 mM TEAB pH 8.5, as mobile phase A, and 72 mM TEAB in ACN pH 8.5, as mobile phase B (10 min with 2% mobile phase B followed by a linear gradient until 45% mobile phase B during 60 min, then followed by column wash and re-equilibration). Throughout the run, 74 fractions were collected, which were then joined into 19 samples that were evaporated and prepared for LC-MS/MS analysis. Peptides were resolved by liquid chromatography (nanoLC Ultra 2 D, Eksigent—AB Sciex, Framingham, MA, USA) on a ChromXP^TM^ C18 AR reverse phase column (300 µm ID × 15 cm length, 3 µm particles, 120 Å pore size, Eksigent—AB Sciex, Framingham, MA, USA) at 5 µL/min. Peptides were eluted with an ACN gradient in 0.1% formic acid (2 to 30% ACN, in a linear gradient for 80 min, followed by a column wash and equilibration step), and ionized using an electrospray ionization source (DuoSpray^TM^ Source, AB Sciex, Framingham, MA, USA). The mass spectrometer (Triple TOF^TM^ 5600 System, AB Sciex, Framingham, MA, USA) was programmed for scanning full spectra (350–1250 m/z) for 250 ms, followed by up to 30 MS/MS scans (100–1500 m/z for 100 ms each). Candidate ions with a charge state between +2 and +5 and a minimum threshold of 70 counts/s were isolated for fragmentation and two MS/MS spectra were collected, before adding those ions to the exclusion list for 15 s (the mass spectrometer was operated by Analyst TF 1.6, AB Sciex, Framingham, MA, USA). Specific iTRAQ rolling collision energy was used.

Peptide and protein identification and quantification were performed with ProteinPilot™ (v4.5, AB Sciex, Framingham, MA, USA). The search parameters used were the following: SwissProt database (release 2012_06) using *Homo sapiens* proteins, and MMTS alkylated cysteines and iTRAQ labeled peptides were set as fixed modifications. For data normalization, both Protein Pilot’s bias and background corrections were performed. The first allows for correcting systematic errors due to unequal mixing of labeled samples, by calculating the median protein ratio for all proteins reported in each sample, adjusted to unity, and assigning an autobias factor to it. An independent False Discovery Rate (FDR) analysis using the target-decoy approach provided with ProteinPilot software was used to assess the quality of the identifications. Positive identifications were considered when identified proteins and peptides reached a confidence value >95% [30] (5% local FDR), corresponding to a threshold cut-off of 2.01 (unused ProtScore). To increase the confidence level of quantified proteins, only those with at least 2 peptides used for quantification were considered for further comparative analysis.

The analysis of differentially expressed proteins was performed as follows. The Benjamini-Hochberg procedure [31] was applied to control the false discovery rate at the protein identification level, using the Protein Pilot *p* values, and a 5% allowed FDR. Using the Protein Pilot-calculated ratios, outliers were identified by computing the standard score (Z score) for the Protein Pilot values for each identified protein. When Z score values were more than 1 standard deviation away from the mean values, the corresponding Protein Pilot ratio was considered an outlier and removed. For each identified protein, fold change (FC) was computed from the remaining values as the base 2 logarithm of the remaining Protein Pilot ratios, with an associated Fisher’s combined *p* value [32,33]. Proteins were considered significantly different between samples when fold-change was equal to or greater than 1.5, at a significance level of 0.05.

Data were analyzed with different bioinformatics tools. Enrichment analysis for the gene ontology categories was performed using g:Profile online tool [34] and DAVID [35] (on 22 October 2022), using the whole human genome as a reference. Data mining was performed using VOSviewer (on 3 June 2022).

### 2.9. Western Blot Analysis

For the evaluation of radiation-induced DNA damage, H2AX phosphorylation was analyzed by Western blot. Briefly, macrophage proteins were extracted with Laemmli buffer 1× (3% glycerol, 5% β-mercaptoethanol, 2% SDS, 0.1% blue bromophenol in 1 M Tris-HCl pH 6.8), about 40 min after irradiation (5 × 2 Gy). Macrophage lysates (*n* = 3) were sonicated for 5 s to shear DNA and, heated at 95 °C for 5 min, and 5 μg was loaded on 15% SDS-polyacrylamide gels. Proteins were separated by electrophoresis and transferred onto nitrocellulose membrane (GE Healthcare, Chicago, IL, USA). Membranes were blocked in 5% powered milk, diluted in PBS-Tween 0.5%, for 1 h. Incubation with primary antibody against histone-H2AX (Ser139) (γH2AX) (clone JBW301) (Millipore, Burlington, MA, USA) was performed overnight at 4 °C. Antibody against α-tubulin (Sigma-Aldrich, St. Louis, MO, USA) was used to normalize protein loading. Sheep anti-mouse horseradish peroxidase (HRP)-conjugated secondary antibody (Amersham—GE Healthcare, Chicago, IL, USA) was used for 1 h, at RT, followed by ECL detection (GE Healthcare, Chicago, IL, USA).

To evaluate the protein expression of other targets of interest, macrophage proteins were extracted with RIPA buffer, composed as described above for proteomics, at 24 h after IR (5 × 2 Gy) exposure. About 40 µg (for ferritin and ferroportin) or 25 µg (for all the other targets) of protein was diluted in Laemmli buffer containing β-mercaptoethanol (BioRad), denatured at 95 °C and loaded in SDS-polyacrylamide gels. Primary antibodies against cathepsin D (clone BC011, Millipore; clone H68.4, Thermo Fisher, Waltham, MA, USA), transferrin receptor protein 1 (Novocastra—Leica Biosystems, Carnaxide, Portugal), DMT1 (ProteinTech, Manchester, UK), ferroportin (Novus Biologicals, Centennial, CO, USA) or ferritin (GeneTex, Irvine, CA, USA) were used. Protein bands were quantified using Quantity One (version 4.6.5, BioRad, Hercules, CA, USA) or ImageJ software (version 1.52 a) [36].

### 2.10. Animal Experiments

Mouse tumors were established and irradiated, as previously reported by our group [37]. Briefly, 4-week-old immunocompetent BALB/cByJ females (Charles River Laboratories, Wilmington, MA, USA) were injected orthotopically in the mammary fat pad with 1 × 10^6^ 4 T1-luciferase cells and tumor progression was followed by bioluminescence imaging. A week later, the tumor was exposed (IR, *n* = 6 animals) or not (Ctr, *n* = 6 animals) to 2 ionizing radiation fractions of 5 Gy each (2 × 5 Gy), using a Small Animal Research Radiation Platform (SARRP) (X-ray tube: ISOVOLT 225 M2 X-ray source; SARRP system, XStrahl^®^, Walsall, UK), at a constant rate of 2.83 Gy/min, for 106 s. The voltage of the X-ray source was fixed at 220 kV with a tube current of 13 mA, emitted from the 2.5 mm focal spot and filtered by a copper filter of 0.15 mm and a 5 mm × 5 mm collimator, which reduces the irradiation field to the desired target size. The SARRP allows highly localized irradiation on small animals. At day 28 after cancer cell inoculation, i.e., 18 days after irradiation, the animals were sacrificed, and tumors were removed for further analysis.

### 2.11. Immunohistochemistry for TfR1

Immunohistochemistry (IHC) was used for the detection of TfR1 levels in macrophages, from irradiated (*n* = 6) and non-irradiated (*n* = 6) mouse breast cancer tumor samples. Paraffin blocks were sectioned in 3 μm slices and sequential stainings for hematoxylin and eosin (H&E), F4/80 (macrophage lineage marker) and TfR1 were performed (3–6 tumor sections per animal).

Tissues were deparaffinized, hydrated and antigen retrieval was performed in Citrate Buffffer (10 mM, pH = 6) at 95 °C for 25 min (TfR1) or in Proteinase K (20 µg/mL in Tris-EDTA-CaCl_2_ Buffer, pH = 8) at 37 °C for 20 min (F4/80). Endogenous peroxidase activity was blocked with 3% hydrogen peroxide in methanol (*v*/*v*) for 15 min at (RT) in the dark. To block endogenous immunoglobulins, mouse-on-mouse blocking was performed for 30 (TfR1) or 60 (F4/80) min at RT, using AffiniPure Fab Fragment Goat Anti-Mouse IgG (H+L) (1:50 in 5% Bovine Serum Albumin (BSA), Jackson Immuno Research, West Grove, PA, USA). Non-specific binding was blocked using UltraVision Protein Block (Thermo Fisher) for 30 min at RT (TfR1) or 1:5 Goat Serum Albumin (GSA) in 10% BSA for 1 h (F4/80) at RT. Slides were incubated with primary antibodies ON at 4 °C: mouse monoclonal anti-TfR1 (1:50, clone H68.4, ThermoFisher, Waltham, MA, USA) or rat monoclonal anti-F4/80 (1:50, clone BM8, BioLegend, San Diego, CA, USA). After washing, incubation with secondary horseradish peroxidase (HRP)-conjugated antibodies was performed for 1 h at RT in the dark: sheep anti-mouse (1:200, NA931, Cytiva, Sigma-Aldrich, St. Louis, MO, USA) was used against the TfR1 antibody and goat anti-rat (1:300, sc-2006, Santa Cruz Biotechnology, Dallas, TX, USA) against the F4/80 antibody. Peroxidase activity was detected using diaminobenzidine (DAB) solution (Sigma-Aldrich, St. Louis, MO, USA) and 30% hydrogen peroxide. Importantly, DAB incubation time was maintained for all slides. Sections were counterstained with hematoxylin, dehydrated and mounted in Entellan^TM^ (Merck Millipore, Burlington, MA, USA).

The slides were digitalized using NanoZoomer 2.0 HT (Hamamatsu, Shizuoka, Japan) and visualized using the Slidex software (DMA-IPATIMUP, version 1.1). Microscopic fields of peri-tumoral tissue were acquired (200 × total magnification-) in both TfR1 and F4/80 images of each animal specimen and semi-quantification of TfR1 signal was performed in a minimum of 100 macrophages per animal, using the ImageJ/Fiji software154 (version 1.52 a). Paired images of the same area stained for TfR1 or F4/80 were aligned using the BigWarp plugin. F4/80 images were color deconvoluted using the H DAB vector, the threshold of the DAB channel was adjusted, and Regions of Interest (ROIs) were defined for F4/80^+^ cells. These ROIs were then overlaid over the TfR1 warped images, manually adjusted, and DAB chromogen intensity was measured and correlated to TfR1 expression.

### 2.12. Statistical Analysis

All graphs and statistical analysis were performed using GraphPad Prism Software v5 (GraphPad-trial version). Data were tested for normality using the D’Agostino-Pearson omnibus. If data were normal, the parametric one sample or paired *t*-tests were used to test the hypothesis that irradiated macrophages were different from non-irradiated ones. If data were not normal, the non-parametric Wilcoxon signed-rank test was applied. Statistical significance was achieved when *p* < 0.05. The number of independent experiments performed as well as the number of animals or macrophage donors used, which correspond to biological replicates, are indicated in the legend of each figure.

## 3. Results

### 3.1. Despite DNA Damage, Irradiated Macrophages Remain Viable

**Figure 1:**
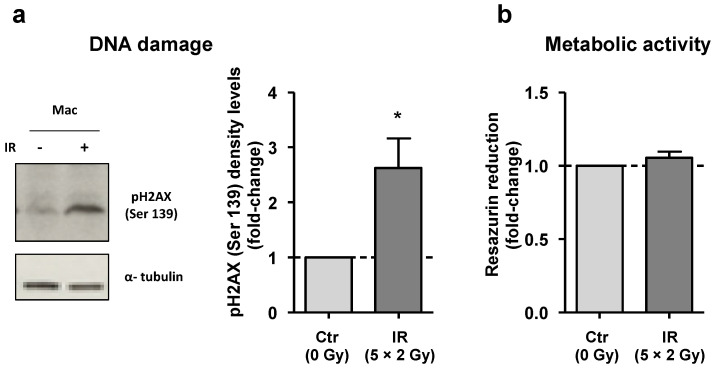
Despite DNA damage, irradiated macrophages remain metabolically viable. (**a**) DNA damage induced by cumulative ionizing radiation doses (5 × 2 Gy) in macrophages was confirmed by Western blot analysis for phosphorylated H2AX (Ser139) (ɤH2AX), 40 min after exposure to the last irradiation dose. Quantification of ɤH2AX band intensity of irradiated macrophages, normalized to the control, is represented in the graph (*n* = 3). (**b**) The metabolic activity of irradiated macrophages (*n* = 8) was measured using the resazurin reduction assay and normalized to that of non-irradiated ones. Mean ± SEM is presented. The whole blots (uncropped blots) showing all bands with all molecular weight markers on the Western are provided in the Supplementary Materials. * *p* < 0.05.

To determine whether the fractionated irradiation protocol used caused significative DNA damage, the phosphorylation level of histone H2AX (Ser139) (ɤH2AX), a sensitive marker of DNA double-strand breaks [28], was evaluated at 40 min after last irradiation dose by Western blot analysis (Figure 1a and Figure S1). Protein band quantification demonstrated that irradiated macrophages have, on average, 2.5 times more phosphorylated H2AX than non-irradiated ones. To determine whether macrophages remained viable after irradiation, we performed the resazurin reduction assay, which is considered a simple and non-destructive method to measure cell response (namely cytotoxicity) to irradiation [29]. Results revealed that despite the DNA damage, macrophages remained viable (Figure 1b), thereby confirming our previous data [23].

### 3.2. IR Interferes with Macrophage Metabolism and Regulation of Transport

To assess the effect of cumulative and clinically relevant IR doses (5 × 2 Gy) on human macrophages, the proteomes of irradiated and non-irradiated macrophages were compared via a gel-free, mass spectrometry-based approach, iTRAQ followed by 2 D-LC. Protein expression was analyzed at 24 h post-irradiation instead of immediately after irradiation to avoid detection of acute radiation effects. A total of 1343 protein groups were confidently identified (*p* ≤ 0.05) (Supplementary Tables S1 and S2), of which 1117 (proteins with at least two peptides) were further used for quantification (Supplementary Tables S3 and S4). After removal of outlier values for each protein target (Supplementary Table S5), a statistical analysis evidenced the existence of 27 differentially expressed targets (*p* ≤ 0.05 and fold-change above 1.5) between irradiated and non-irradiated macrophages. A functional enrichment analysis demonstrated that these targets are mostly associated with cell metabolism (NADPH and glyceraldehyde-3-phosphate metabolic processes, and regulation of fatty acid biosynthetic process), regulation of localization (refers to any process in which a cell, a substance, or a cellular entity is transported to, or maintained in, a specific location), and regulation of transport (early endosome to late endosome transport) (Figure S2). Regarding molecular function, they are associated with cell adhesion molecule binding, transketolase or transaldolase activity, and RNA binding. In terms of cellular components, these targets were mainly associated with extracellular exosomes. Overall, the pentose phosphate metabolism (a metabolic pathway parallel to glycolysis) is deregulated (Figure S2).

From the 27 statistically deregulated targets, we selected those that exhibit the same trend in at least 3 donors, obtaining 21 targets. By applying this criterion, we hypothesized to obtain a list of the most strongly deregulated targets, possibly related with higher biological relevance. We found 19 downregulated proteins, in contrast to only 2 upregulated proteins in irradiated macrophages (Table 1).

### 3.3. Cathepsin D, a Positive Regulator of Apoptosis, Is Downregulated in Irradiated Macrophages

**Figure 2:**
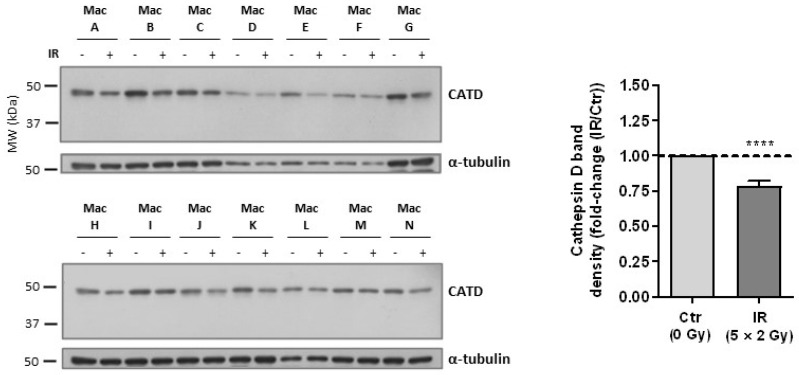
Validation of cathepsin D downregulation in irradiated macrophages (5 × 2 Gy), compared with non-irradiated controls. Western blot analysis of the same donors used for iTRAQ (*n* = 4, Mac A–D) and of additional donors (*n* = 10, Mac E–N). The graph depicts the quantification of cathepsin D band intensity, normalized to α-tubulin staining (fold-change: IR/Ctr). Data are represented as the mean ± SEM (**** *p* < 0.0001). A one-sample *t*-test was used for statistical analysis. The whole blots (uncropped blots) showing all bands with all molecular weight markers on the Western are provided in the Supplementary Materials.

Given that we have previously found that irradiated macrophages did not enter into apoptosis [23], we are now particularly interested on identifying proteins that may explain this radiation resistant phenotype. One of the most strongly downregulated targets in irradiated macrophages, obtained in the present iTRAQ dataset, was cathepsin D (average ratio of 0.4, indicating a decreased expression of about 2.5×), which is a lysosomal marker [38] and a positive regulator of apoptosis. We validated the reduced expression of cathepsin D by Western blot in the same donors used for iTRAQ (Mac D-G), and in a distinct subset of 10 donors, which were all considered biological replicates (Figure 2 and Figure S3).

Cathepsin D is synthesized in the rough endoplasmic reticulum as preprocathepsin D, cleaved into inactive procathepsin D, and then subjected to post-translational modification events and transported to the Golgi apparatus, where it acquires the recognition signal for endosomal/lysosomal transport [39,40]. After being converted into the active single-chain molecule (48 KDa) in lysosomes, cathepsin D is further processed into a mature two-chain form [41]. Thus, the identified cathepsin D band, which appears below 50 KDa, most likely corresponds to the active single-chain molecule (48 KDa) located in lysosomes. 

### 3.4. Irradiated Macrophages Exhibit a Reduction of Both Glucose Uptake and Total ATP Levels

**Figure 3:**
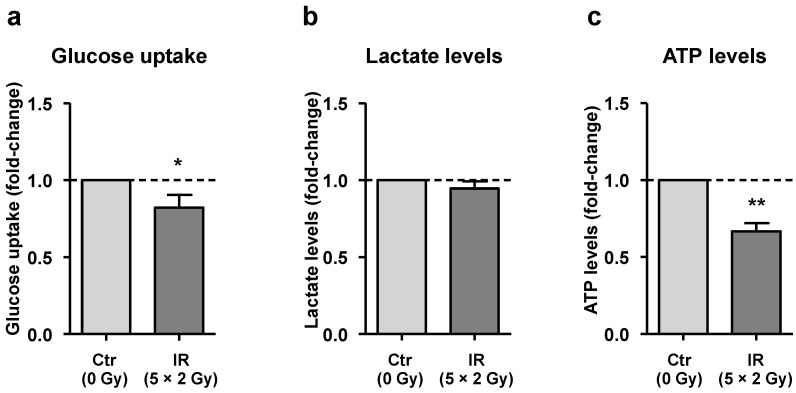
IR affects macrophage metabolism by promoting a reduction of both glucose uptake and total cellular ATP levels. (**a**,**b**) Both glucose (*n* = 12) and lactate (*n* = 14) levels were determined in conditioned medium (CM) of irradiated (IR, 5 × 2 Gy) and non-irradiated (Ctr, 0 Gy) macrophages. To estimate glucose uptake, glucose levels were subtracted to the initial glucose concentration of RPMI medium. (**c**) ATP was measured in irradiated (IR, 5 × 2 Gy) and non-irradiated (Ctr, 0 Gy) macrophages after cell lysis (*n* = 6). Data are represented as the mean ± SEM (* *p* < 0.05 and ** *p* < 0.01). A one sample *t*-test was used for statistical analysis.

Our iTRAQ dataset suggests that irradiated macrophages exhibit decreased expression of D-3-phosphoglycerate dehydrogenase, which converts d-3-phosphoglycerate (PGA), an intermediate in glycolysis, to phosphohydroxypyruvate (PHP) concomitant with the reduction of NAD^+^ (first step of L-serine biosynthesis pathway) [42]. To find out whether lower enzyme levels could be justified by less substrate resulting from downregulated glycolysis, glucose and lactate (a product of glycolysis) levels were evaluated in macrophage CM. Results demonstrated that IR exposure caused a significant increase in glucose levels in macrophage CM, supporting a decrease in glucose uptake (Figure 3a), without significantly affecting lactate levels (Figure 3b).

Since a decrease in glucose uptake could result in lower ATP production, we compared total ATP levels in irradiated and non-irradiated macrophages. Indeed, we found a significant decrease in total ATP levels in irradiated macrophages when compared with the non-irradiated ones (Figure 3c).

However, it is important to note that ATP levels result from the balance between ATP production and consumption. While ATP is mainly produced from complete glucose oxidation (consisting of glycolysis, citric acid cycle and oxidative phosphorylation), it is consumed by protein synthesis and Na^+^/K^+^ ATPases, which catalyze the hydrolysis of ATP coupled with the exchange of Na^+^ and K^+^ ions across the plasma membrane [43,44,45,46]. Interestingly, our iTRAQ data also demonstrated that the [Na^+^/K^+^]-transporting ATPase subunit α-1 (encoded by the *ATP1 A1* gene), which is an isoform of the Na^+^/K^+^ ATPase catalytic subunit, was upregulated in irradiated macrophages. From all statistically significant deregulated targets, this was the only one that was consistently altered in all 4 donors (average ratio of 1.98). These data suggest that the lower ATP levels observed in irradiated macrophages could be the combination of lower ATP production, resulting from lower glucose uptake, and higher consumption, through increased expression of Na^+^/K^+^ ATPase subunit α-1.

### 3.5. Transferrin Receptor Protein 1 (TfR1 or CD71), a Receptor Specialized in Cellular Iron Uptake, Is Upregulated in Irradiated Macrophages

**Figure 4:**
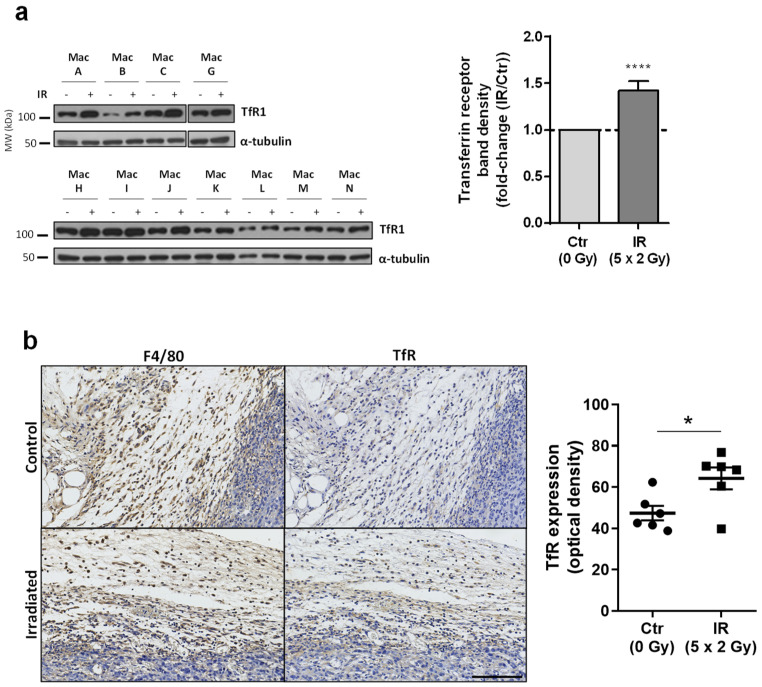
TfR1 is upregulated in macrophages irradiated in vitro and in vivo. (**a**) TfR1 expression was evaluated by Western blot in irradiated macrophages and compared with non-irradiated ones (*n* = 11). The graph depicts the quantification of TfR1 band intensity, normalized to α-tubulin staining. Data are presented as the mean ± SEM (**** *p* < 0.0001). A one-sample *t*-test was used for statistical analysis. (**b**) Representative images of immunohistochemistry for F4/80 (macrophage marker) and TfR1 in irradiated and non-irradiated mouse breast peritumoral tissue. Scale bar = 100 µm. Semi-quantitative analysis of macrophage TfR1 expression in irradiated (IR, *n* = 6) and non-irradiated (Ctr, *n* = 6) animals is depicted. Values represent the mean optical density of TfR1 staining of a minimum of 100 macrophages per animal. Data is presented as the mean ± SEM (* *p* < 0.05) and each symbol represents one animal. A one-sample *t*-test was used for statistical analysis. The whole blots (uncropped blots) showing all bands with all molecular weight markers on the Western are provided in the Supplementary Materials.

To find additional molecules potentially affected by IR exposure in macrophages, we performed a data mining analysis. The automatic text-mining functionality of VOSviewer was used to retrieve and create co-occurrence networks of terms associated with macrophage and radiation or X-rays or radiotherapy. The respective literature search yielded 1440 articles (on 3 June 2022), which resulted in a list of 1111 terms. From this, several terms emerged, namely a link between apoptosis and iron (Figure S4). Given the particular importance of macrophages in iron metabolism and of iron on immune cell function [47,48], we pursued this clue by first evaluating the protein expression of the transferrin receptor (TfR1, also known as CD71), which is responsible for the uptake of transferrin-bound iron, the most common form of iron in the blood, through receptor-mediated endocytosis. The expression of TfR1 was found to be significantly upregulated in irradiated macrophages (Figure 4a and Figure S3).

To evaluate the relevance of the IR-induced macrophage TfR1 upregulation in vivo, we measured TfR1 expression in tumor-associated macrophages from tumor sections obtained from an animal experiment previously published by our group [37]. Briefly, BALB/c mice were injected with 4 T1-Luciferase cells (triple-negative breast cancer cells) on the mammary fat pad and submitted to radiotherapy (2 × 5 Gy). Due to logistic issues related with animal irradiation, we had to reduce the number of IR daily fractions to 2 instead of the 5 used in the in vitro study, and consequently, to maintain the same cumulative IR dose (10 Gy) applied in vitro, the dose per fraction increased (5 Gy instead of 2 Gy). In fact, with this alteration we mimicked 2 days of the hipofractionated radiotherapy scheme of 5 × 5 Gy started to be used one decade ago for locally advanced rectal cancer [49].

Regarding tumor model characterization, we have previously shown that the tumor size at the irradiation time was 123 ± 12 mm^3^ [37]. Six days after irradiation, the tumor growth was significantly reduced (Figure S5A) and immediately before the experimental endpoint (at 18 days post-IR, i.e., 28 days post-4 T1 injection), irradiated animals still exhibited a delayed primary tumor growth (Figure S5B), with statistically significant reduction in tumor weight (0.4 ± 0.1 g; *p* < 0.05) [37]. Additionally, IR reduced the metastatic score in the lungs, which means smaller metastases than the animals from control group [37].

TfR1 and F4/80 (mouse macrophage marker) immunohistochemistry staining of mouse tumor sections evidenced that macrophages from animals exposed to IR exhibit a statistically significant increased expression of TfR1 when compared to non-irradiated macrophages (Figure 4b). This suggests that the IR-induced macrophage TfR1 upregulation observed in vitro is translatable into a more complex in vivo context, such as the tumor microenvironment. Similar to what we observed in macrophages from human donors, a high variability in TfR1 expression was observed in macrophages from different animals, particularly in the irradiated group.

### 3.6. Macrophage Iron Metabolism Is Affected by IR Exposure

**Figure 5:**
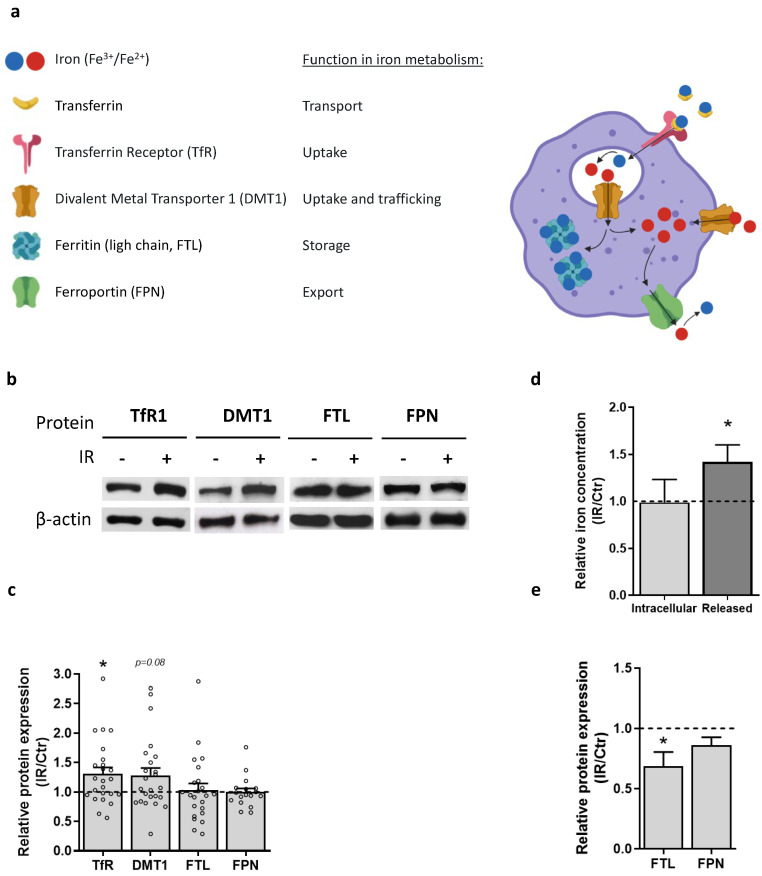
IR affects macrophage iron metabolism. (**a**) Simplified schematic representation of iron trafficking in macrophages, highlighting the proteins analyzed herein by Western Blot. The image was created using BioRender. (**b**) Representative image of Western Blot analysis of iron metabolism-associated proteins in irradiated (IR, 5 × 2 Gy) and non-irradiated (Ctr) macrophages. β-actin was used as a loading control. The graph indicates the relative (IR/Ctr) protein expression of TfR, DMT1, FTL, and FPN in macrophages. Data were obtained from 5 independent irradiation experiments (*n* = 17–24 macrophage donors) and are presented as the mean ± SEM (* *p* ≤ 0.05). The Wilcoxon signed-rank test was used for statistical analysis. (**c**) Graph depicting the quantification of intracellular and released iron in irradiated (IR, 5 × 2 Gy) and non-irradiated (Ctr) macrophages. Values are represented as IR/Ctr ratios, normalized to cell number. Data were obtained from one irradiation experiment for intracellular iron (*n* = 6) and two independent irradiation experiments for released iron (*n* = 12), and is presented as the mean ± SEM (* *p* ≤ 0.05). A one-sample *t*-test was used for statistical analysis. (**d**) Quantification of intracellular and released iron in irradiated (IR, 5 × 2 Gy) and non-irradiated (Ctr) macrophages, measured through Inductively Coupled Plasm–Atomic Emission Spectroscopy (ICP-AES). Values are represented as IR/CT ratios. Data were obtained from 1 irradiation experiment for intracellular iron (*n* = 6 macrophage donors) and 2 independent irradiation experiments for released iron (*n* = 12 macrophage donors) and is presented as the mean ± SEM (* *p* ≤ 0.05). A one sample *t*-test was used for statistical analysis. (**e**) Relative protein expression of FTL and FPN in macrophages from selected donors with increased iron release, represented through IR/Ctr ratios, analyzed by Western blot. β-actin was used as a loading control. Data were obtained from 2 independent irradiation experiments (*n* = 7) and is presented as the mean ± SEM (* *p* ≤ 0.05). A one *t*-test was used for statistical analysis. The whole blots (uncropped blots) showing all bands with all molecular weight markers on the Western are provided in the Supplementary Materials.

The discovery of increased TfR1 expression in irradiated macrophages suggests higher uptake of transferrin-bound iron. To further investigate whether IR could alter macrophage iron metabolism, we evaluated the expression of the following iron metabolism-associated proteins, in a new set of macrophage donors: divalent metal transporter 1 (DMT1), which transports iron across the endosomal membrane to the cytosol; ferritin light chain (FTL), a crucial molecule for intracellular iron storage; and ferroportin (FPN), the only known cellular iron exporter (Figure 5a). The expression of these proteins provides us an overall picture of macrophage iron metabolism, regarding iron uptake, trafficking, storage, and export. Results show that together with TfR1 upregulation, IR tends to increase DMT1 expression, suggesting the mobilization of iron from the endosome into the cytosol, without affecting neither FTL nor FPN expression (Figure 5b,c and Figure S6). This means that higher iron uptake in irradiated macrophages does not seem to be followed by a rise in iron storage nor in FPN-dependent export. 

To further evaluate if radiation affects macrophage iron metabolism, we measured intracellular iron levels and iron release into the culture medium of irradiated cells and non-irradiated controls. In agreement with the lack of changes in FTL expression, we found no differences in the intracellular iron levels of irradiated and non-irradiated macrophages. However, upon supplementation of the culture medium with an iron source (ferric ammonium citrate), IR led to a significant increase in iron release from macrophages into the culture medium (Figure 5d). This was somehow unexpected given that IR did not induce expression of FPN, the only known cellular iron exporter. Therefore, we analyzed protein expression in macrophages from a selected set of donors, which exhibited increased levels of released iron (fold-change > 1.25). Interestingly, in this set of donors we observed a significant decrease in FTL expression upon irradiation, while FPN expression remained unaltered, consistent with the activation of an FPN-independent iron export mechanism (Figure 5e). These results suggest that in irradiated macrophages most of the internalized iron is not being stored, but likely incorporated into the labile intracellular pool, remaining highly bioavailable for rapid export, through a potential FPN-independent mechanism. Altogether, irradiated macrophages seem to acquire an iron recycling phenotype, characterized by increased iron uptake and release.

## 4. Discussion

In this work, we have studied the effect of radiotherapy on human macrophages, adding new data in the field and complementing our previous study [23]. Contrary to the majority of the studies, we have exposed macrophages to clinically relevant IR doses, mimicking one week of a cancer patient´s treatment, using a fractionated scheme of 2 Gy/fraction/day for 5 days, rather than low or single IR doses. We compared the protein expression profile of irradiated versus non-irradiated macrophages to identify a radiation-induced molecular signature, which would contribute to better understanding of macrophage radioresistance. 

Our results evidenced that macrophages irradiated in vitro exhibit alterations in cell metabolism and regulation of transport. We validated the downregulation of one of the most strongly deregulated targets—cathepsin D, an abundant lysosomal protease. We also tried to validate cathepsin D expression in vivo, but we were not able to optimize the immunohistochemistry protocol due to a persistent lack of specific antibody signal. Besides lysosomes, cathepsin D is also present in phagosomes, which are structures responsible for the engulfment of bacteria and other particles, and in endosomes [39,50,51]. Based on its ability to cleave a wide range of substrates, cathepsin D is involved in numerous physiological functions, namely protein degradation in the acidic milieu of lysosomes, antigen processing and regulation of programmed cell death [52]. In general, the release and diffusion of cathepsins and other hydrolases from the lysosomal lumen to the cytosol leads to the degradation of vital proteins and causes damage to other cellular components, inducing cell death [53]. Particularly, cathepsin D can activate pro-apoptotic Bid, through specific processing [54], and truncated Bid activates the intrinsic apoptotic pathway by binding to Bax, which leads to mitochondrial outer membrane permeabilization and consequent cytochrome c release [55]. Cathepsin D activation was shown to trigger apoptosis during pneumococcal infection in macrophages, while its pharmacological inhibition blocked this process [56]. Unlike macrophages, which we have previously demonstrated not to undergo apoptosis after IR exposure [23], breast cancer cells exhibit increased cathepsin D expression and significant reduction of viability, upon exposure to single (10 Gy) or fractionated (5 × 2 Gy) radiotherapy doses [57]. Altogether, this suggests a relation between low cathepsin levels and protection from radiation-induced apoptosis in macrophages. This hypothesis is supported by the reported role of RelA/NF-κB transcriptional activation in protecting cells from the lysosomal pathway of cell death [58]. Along this line, RelB (non-canonical NF-κB activation) overexpression in breast cancer cells induced cathepsin D downregulation [59]. According to this evidence, our previous work demonstrated that irradiated macrophages exhibited an increase in NF-κB transcriptional activation (particularly of RelB subunit), which together with increased Bcl-xL expression, may promote macrophage survival after IR exposure [23].

Macrophages are major regulators of iron homeostasis due to their ability to recycle iron. It is well established that iron is vital for cell division, growth, and survival, and that malignant cells present an even higher requirement for iron [60]. The essential role of macrophages in iron recycling is well-known and it has been reported that tumor-associated macrophages often acquire an iron-donor phenotype, serving as a source of iron for tumor cells, promoting tumor cell growth and progression [61,62]. Accordingly, CM from M2 macrophages was shown to significantly enhance tumor cell proliferation, an effect that could be blunted by iron chelation [61,62]. Particularly TfR1 allows for the uptake of transferrin-bound iron through receptor-mediated endocytosis, after which iron dissociates from TfR1 within acidified endosomes. Through this study, we demonstrated that TfR1 was upregulated in macrophages irradiated in vitro and in vivo, from an orthotopic triple-negative breast cancer mouse model, whose tumor was irradiated through the precise SARRP system. Importantly, TfR1 expression was evaluated at a later time-point in the in vivo study when compared with the in vitro one (s vs. 24 h post-irradiation), suggesting that the elevated TfR1 expression can be sustained for a long time after irradiation and may have an impact in treatment outcome. Nevertheless, the same cumulative IR dose (10 Gy), although achieved through different fractionated schemes (5 × 2 Gy for in vitro experiments and 2 × 5 Gy for the in vivo ones), produced the same result, reinforcing TfR1 deregulation in macrophages upon IR exposure.

In macrophages, TfR1 is often upregulated during bacterial infection, being crucial for the proliferation of some intracellular pathogens [63]. Regarding the effect of IR on TfR1 levels, it was reported that some splenic mononuclear cell populations exhibited upregulation of transferrin receptors following mice whole-body irradiation [64]. In human cells, higher TfR1 expression was found in far-ultraviolet (UV) light-resistant cells rather than in UV-sensitive ones [65]. Accordingly, depletion of TfR reduced UV-resistance, while overexpression increased it, which was suggested to be associated with an anti-apoptotic effect of this growth factor receptor [65]. Interestingly, Trf1 depletion avoids NF-κB nuclear translocation and its consequent activation, and increases apoptosis in response to TNF-α [66].

Following TfR1 expression change, we further explored how IR affects macrophage iron metabolism. Results demonstrated that irradiated macrophages acquire what seems to be an iron-recycling phenotype, characterized by increased iron uptake and its mobilization into the cytosol, demonstrated by elevated TfR1 expression and a tendency for higher DMT1 protein levels. DMT1 is localized in the endosomal membrane, being involved in iron mobilization from the endosome to the cytosol, and in the cell membrane, where it contributes to the uptake of non-transferrin-bound iron (NTBI). The FBS present in the culture medium is known to contain high levels of transferrin-bound iron, but not NTBI, much like the blood. Consequently, it is unlikely that macrophages present high levels of membrane-localized DMT1 in our experimental setup. However, further experiments are required to confirm our hypothesis that the apparent increase in DMT1 expression is associated with the upregulation of TfR1 and is likely contributing to the mobilization of iron from the endosome into the cytosol.

Irradiated macrophages also exhibit increased iron release, as shown by increased iron concentration in the CM after IR exposure. Importantly, IR-mediated iron release is not associated with increased FPN expression. Further research is required to elucidate the alternative mechanisms of iron export. Importantly, this IR-induced macrophage iron-donor phenotype may potentially contribute to tumorigenesis and radioresistance of cancer cells.

**Figure 6:**
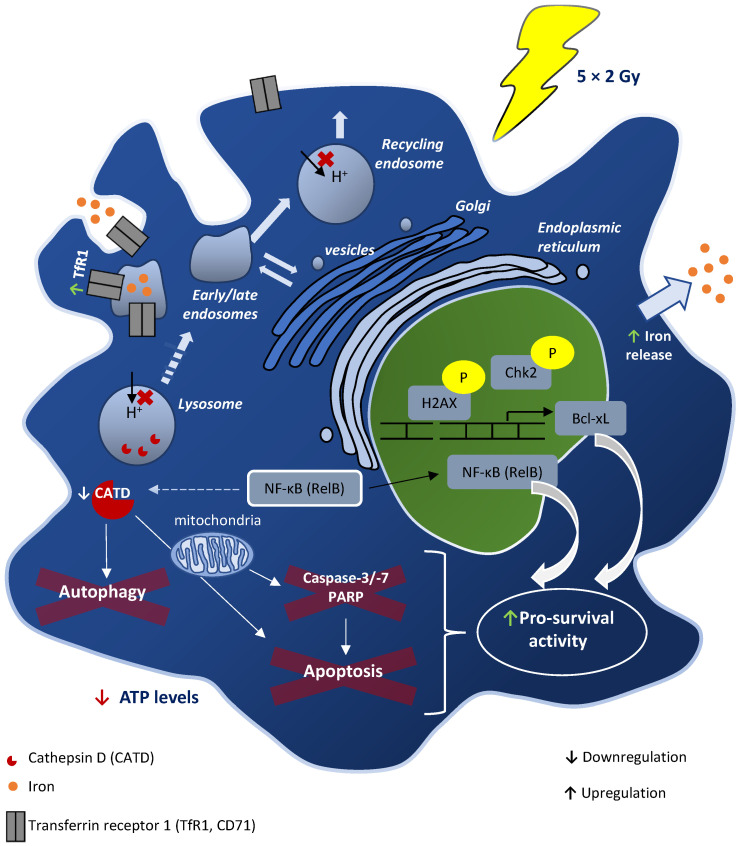
A proposed model for fractionated IR (5 × 2 Gy)-induced effects in human macrophages. This scheme was based on findings obtained from both the present proteomic study and our previous work. We hypothesize that together with increased Bcl-xL expression and RelB nuclear translocation, which we previously described in irradiated macrophages, cathepsin D downregulation could be involved in macrophage survival upon irradiation, by blocking cell death. According to the literature, an association may exist between RelB overexpression and cathepsin D reduction. Additionally, the transferrin receptor, which is involved in iron uptake, was found to be upregulated in irradiated macrophages. Altogether, expression alterations in transferrin receptor and cathepsin D, markers of early-endosomal and lysosome/phagolysosome markers, respectively, may suggest deregulation of these intracellular compartments in irradiated macrophages.

Overall, we speculate that in irradiated macrophages, both cathepsin D downregulation and TfR1 upregulation may be associated with apoptosis suppression and radiation resistance, which could be linked to the previously reported NF-κB RelB subunit activation [23]. Additionally, as TfR1 and mature cathepsin D are considered early-endosomal and lysosome/phagolysosome markers [38], respectively, their deregulated expression in irradiated macrophages may suggest functional alterations in these cellular compartments, supporting a deregulation of transport within the cell (Figure 6). In the future, it would be important to evaluate whether other targets, like Na^+^/K^+^ ATPase subunit α-1, could be also associated with macrophage radioresistance.

## 5. Conclusions

In summary, we presented the first proteomic signature of human macrophages exposed to clinically relevant fractionated IR doses and provided an in vitro and in vivo validation and a global view of the biological processes affected, which can be integrated with our previous published data. The present work increases the general comprehension of macrophage response to IR, providing new insights into the field, and opening new perspectives for further macrophage modulation to improve radiotherapy efficacy. 

## Figures and Tables

**Table 1:** List of differentially deregulated targets between irradiated (5 × 2 Gy) and non-irradiated (0 Gy) macrophages, which exhibit the same trend in at least 3 donors. The main data from the three Gene Ontology categories–biological processes (BP), molecular function (MF) and cellular component (CC) associated with these targets are indicated.

UP Acession Number	Protein	Name	Gene	Average Ratio ^1^	Std	Biological Process	Cellular Comp onent	Molecular Function
P62861	RS30	40 S ribosomal protein S30	*FAU*	0.24	0.11	Cytoplasmic translation, innate immune response in mucosa	Nucleus	Ribonucleoprotein
P29401	TKT	Transketolase	*TKT*	0.33	0.09	Pentose-phosphate shunt	Nucleoplasm, peroxisome, endoplasmic reticulum membrane	Transferase
P67936	TPM4	Tropomyosin alpha-4 chain	*TPM4*	0.35	0.06	Actin filament organization	Cytoskeleton	Actin-binding
P04080	CYTB	Cystatin-B	*CSTB*	0.35	0.24	Negative regulation of proteolysis	Nucleus, Cytoplasm	Protease inhibitor
P07108	ACBP	Acyl-CoA-binding protein	*DBI*	0.36	0.38	Fatty acid metabolic process	Endoplasmic reticulum, Golgi apparatus	Receptor
P02654	APOC1	Apolipoprotein C-I	*APOC1*	0.38	0.34	Lipid transport	Secreted	Fatty acid binding
P07339	CATD	Cathepsin D	*CTSD*	0.40	0.27	Autophagosome assembly, positive regulation of apoptotic process	Lysosome, Secreted	Aspartyl protease
P26038	MOES	Moesin	*MSN*	0.43	0.15	Cytoskeleton organization	Cytoskeleton, Membrane	Actin binding
P11021	GRP78	78 kDa glucose-regulated protein	*HSPA5*	0.52	0.09	Cellular response to unfolded protein	Endoplasmic reticulum	Chaperone, Hydrolase
P04083	ANXA1	Annexin A1	*ANXA1*	0.54	0.21	Immunity, inflammatory response	Membrane, Nucleus, Cytoplasm, Secreted	Phospholipase A2 inhibitor
P63104	1433 Z	14-3-3 protein zeta/delta	*YWHAZ*	0.54	0.11	Negative regulation of apoptosis, protein localization	Cytoplasm	Kinase, Monooxygenase
P19338	NUCL	Nucleolin	*NCL*	0.56	0.17	Negative regulation of translation	Nucleus, Cytoplasm	DNA-binding, RNA-binding
O43175	SERA	D-3-phosphoglycerate dehydrogenase	*PHGDH*	0.59	0.29	Amino-acid biosynthesis	Extracellular exosome	Oxidoreductase
P31948	STIP1	Stress-induced-phosphoprotein 1	*STIP1*	0.59	0.16	Protein folding	Nucleus, Cytoplasm	RNA-binding
Q09666	AHNK	Neuroblast differentiation-associated protein AHNAK	*AHNAK*	0.61	0.25	Regulation of voltage-gated calcium channel activity	Nucleus, Virion	Viral nucleoprotein
Q15149	PLEC	Plectin	*PLEC*	0.62	0.11	Cell morphogenesis	Cytoskeleton	Actin-binding
Q01469	FABP5	Fatty acid-binding protein, epidermal	*FABP5*	0.63	0.12	Lipid transport	Nucleus, Cytoplasm, Secreted	Lipid binding
P21333	FLNA	Filamin-A	*FLNA*	0.63	0.24	Actin cytoskeleton organization	Cytoskeleton	Actin-binding
P30456	1 A43	HLA class I histocompatibility antigen, A-43 alpha chain	*HLA-A*	0.64	0.30	without GO annotation		
Q5 JTZ9	SYAM	Alanine—tRNA ligase, mitochondrial	*AARS2*	1.51	0.66	Protein biosynthesis	Mitochondrion	Aminoacyl-tRNA synthetase
P05023	AT1 A1	Na^+^/K^+^-transporting ATPase subunit alpha-1	*ATP1 A1*	1.80	0.09	Ion transport	Membrane, Cell projection	Translocase

^1^ Average IR/Ctr ratio (from 4 donors). UP: UniProt; Std: standard deviation.

## Data Availability

Proteomics data presented in this study are openly available in [Mendeley Data] at [link 1 10.17632/f8mj5ftjr4.1] and [link 2 10.17632/7xwgk8vykz.1].

## Supplementary Materials

The following supporting information can be downloaded at: https://www.mdpi.com/article/10.3390/cancers15010270/s1, Figure S1: Chemiluminescent/scanned images derived from immunoblotting of macrophages nuclear extracts for γH2AX (Ser 139) 40 min after exposure to 5 × 2 Gy IR doses; Figure S2: Gene Ontology analysis of the proteins differentially expressed between irradiated (5 × 2 Gy) and non-irradiated macrophages; Figure S3: Chemiluminescent/scanned images derived from immunoblotting of macrophages whole-cell lysates for CATD and TfR1 after exposure to 5 × 2 Gy IR doses; Figure S4: Term co-occurrence network of terms associated with macrophage and radiation or X-rays or radiotherapy, evidencing an association between apoptosis and iron; Figure S5: Radiotherapy (2 × 5 Gy) significantly reduced relative tumor growth in 4T1 mouse breast tumor model. Figure S6: Chemiluminescent/scanned images derived from immunoblotting of macrophage whole-cell lysates for TfR1, DMT1, FPN and FTL after exposure to 5 × 2 Gy IR doses. Table S1: List of proteins identified in irradiated (5 × 2 Gy) and non-irradiated macrophages; Table S2: Detailed information on the peptides used to identify proteins; Table S3: List of proteins quantified in irradiated (5 × 2 Gy) and non-irradiated macrophages; Table S4: Detailed information on the peptides used to quantify proteins; Table S5: Detailed information on protein average ratio, after outlier exclusion.
