# Supplementary material for: Macrophage Resistance to Ionizing Radiation Exposure Is Accompanied by Decreased Cathepsin D and Increased Transferrin Receptor 1 Expression
Source: Cancers (Basel). 2022 Dec 30;15(1):270. doi: 10.3390/cancers15010270 (PMC9818572; doi:10.3390/cancers15010270)
Supplement: Supplementary file 1 [file cancers-15-00270-s001.zip › cancers-2037154-supplementary/Supplementary Figures.pdf]

# Macrophage resistance to ionizing radiation exposure is accompanied by decreased Cathepsin D and increased Transferrin receptor 1 expression

**Ana Teresa Pinto[1,2,3*], Ana Beatriz Machado[1,2,4], Hugo Osório[1,5,6], Marta Laranjeira Pinto[1,2], Rui Vitorino[3], Gonçalo Justino[7], Cátia Santa[8,9], Flávia Castro[1,2], Tânia Cruz[1,2], Carla Rodrigues[10], Jorge Lima[1,5], José Luís R Sousa[11], Ana Patrícia Cardoso[1,2], Rita Figueira[12], Armanda Monteiro[12], Margarida Marques[12], Bruno Manadas[9], Jarne Pauwels[13,14], Kris Gevaert[13,14], Marc Mareel[15], Sónia Rocha[16], Tiago Duarte[1,17], Maria José Oliveira[1,2,6]**

[1] i3S - Instituto de Investigação e Inovação em Saúde, Universidade do Porto, *4200-135* Porto, Portugal

[2] INEB - Instituto de Engenharia Biomédica, Universidade do Porto, *4200-135* Porto, Portugal

[3] Department of Medical Sciences, Institute of Biomedicine (iBiMED), Universidade de Aveiro, 3810-193 Aveiro, Portugal

[4] Champalimaud Centre for the Unknown, Fundação Champalimaud, 1400-038 Lisboa, Portugal

[5] IPATIMUP - Instituto de Patologia e Imunologia Molecular da Universidade do Porto, 4200-135 Porto, Portugal

[6] Departament of Pathology, Faculdade de Medicina, Universidade do Porto, 4200-319 Porto, Portugal

[7] Centro de Química Estrutural – Institute of Molecular Sciences, Instituto Superior Técnico, Universidade Técnica de Lisboa, 1049-001 Lisboa, Portugal

[8] CNC - Center for Neuroscience and Cell Biology, Universidade de Coimbra, 3004-504 Coimbra, Portugal

[9] Institute for Interdisciplinary Research (III), Universidade de Coimbra, 3030-789 Coimbra, Portugal

[10] REQUIMTE - LAQV, Chemistry Department, NOVA School of Science and Technology, Universidade de Lisboa, 2829-516 Caparica, Portugal

[11] Personal Health Data Science Group, Sano-Centre for Computational Personalised Medicine, 30-054 Krakow, Poland

[12] Radiotherapy Service, Centro Hospitalar Universitário São João (CHUSJ), EPE, 4200–319 Porto, Portugal

[13] VIB-UGent Center for Medical Biotechnology, 9052 Ghent, Belgium

[14] Department of Biomolecular Medicine, Ghent University, 9052 Ghent, Belgium

[15] Department of Radiation Oncology and Experimental Cancer Research, Ghent University Hospital, 9000 Ghent, Belgium

[16] Institute of System, Molecular and Integrative Biology, University of Liverpool, L69 3GE Liverpool, UK

[17] IBMC - Instituto de Biologia Molecular e Celular, Universidade do Porto, 4200-135 Porto, Portugal

* Correspondence: anapinto@ua.pt

# Supplementary Figures

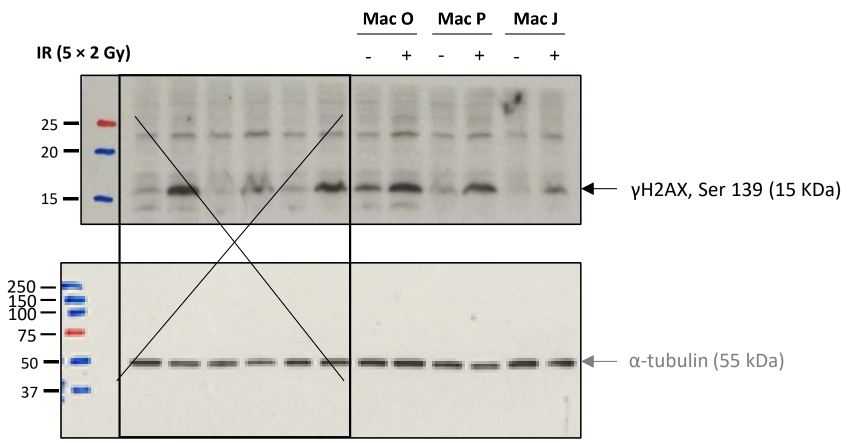


**Figure S1** – Chemiluminescent/scanned images derived from immunoblotting of macrophages nuclear extracts for γH2AX (Ser 139) 40 min after exposure to 5 x 2 Gy IR doses. Loading control: α-tubulin.

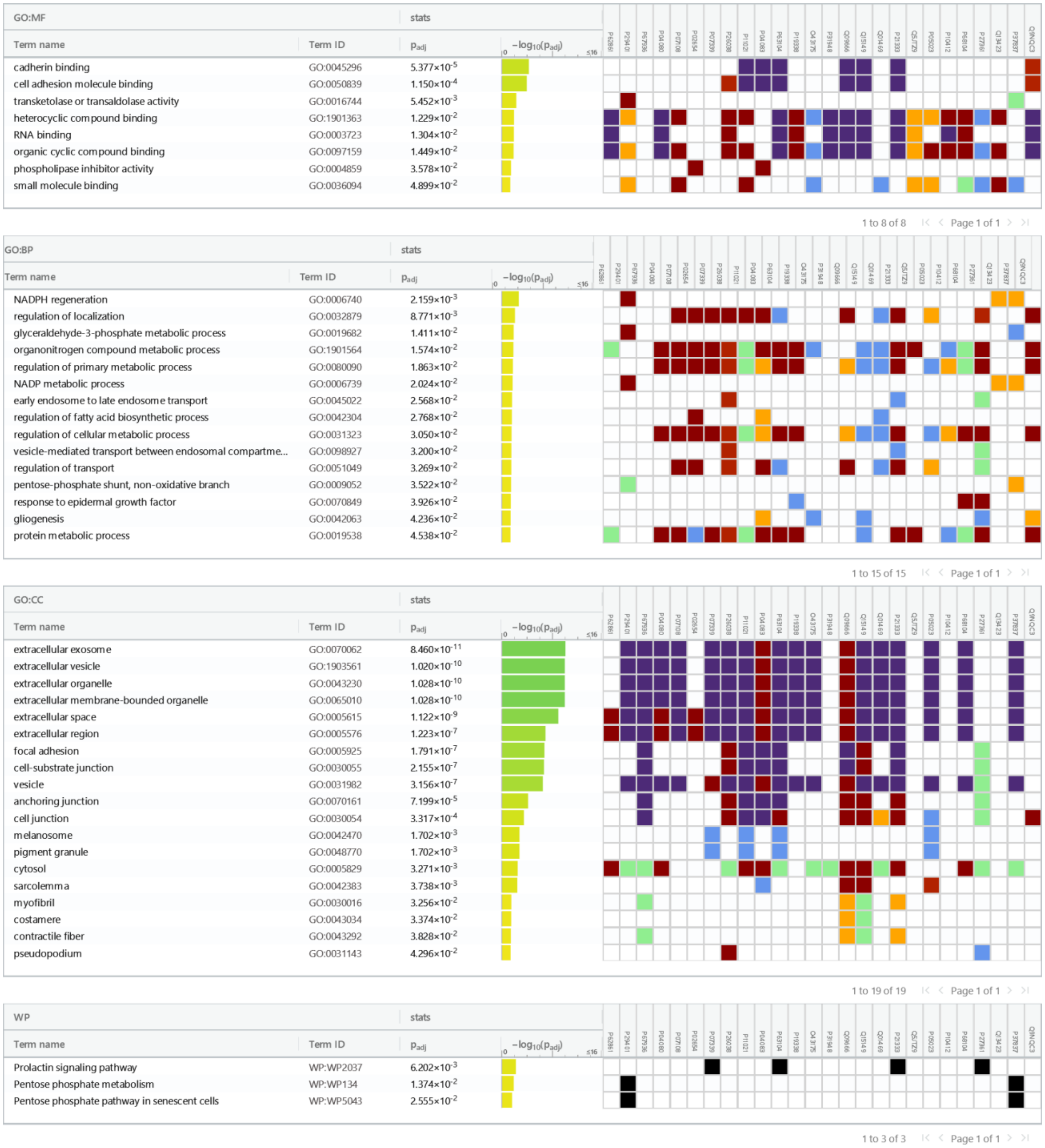


**Figure S2 -** Gene Ontology analysis of the proteins differentially expressed between irradiated (5 x 2 Gy) and non-irradiated macrophages. Data obtained from GProfile (on October 22, 2022). MF: Molecular Function; BP: Biological Process; CC: Cellular Component; WP: WikiPathways. Meaning of the different colors used:

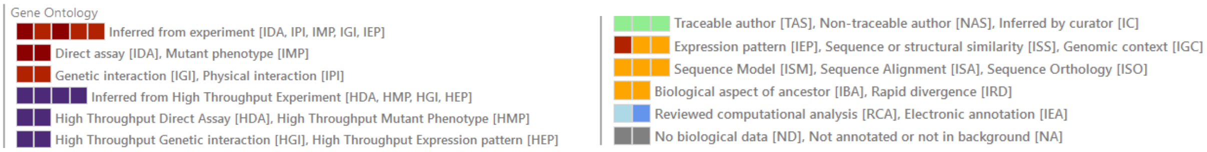

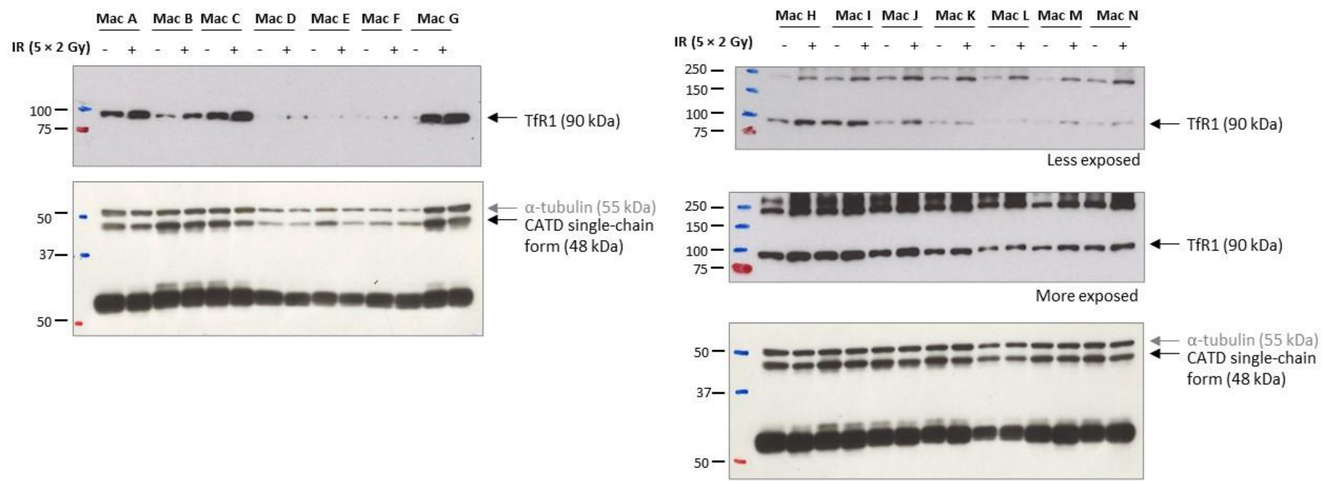


**Figure S3** – Chemiluminescent/scanned images derived from immunoblotting of macrophages whole-cell lysates for CATD and TfR1 after exposure to 5 x 2 Gy IR doses. Membranes were cut between 50 and 75 kDa, and again below 50 kDa, for different antibody staining. Samples from Mac D-F were not considered for TfR1 quantification because insufficient signal was obtained. Loading control: $\alpha$-tubulin.

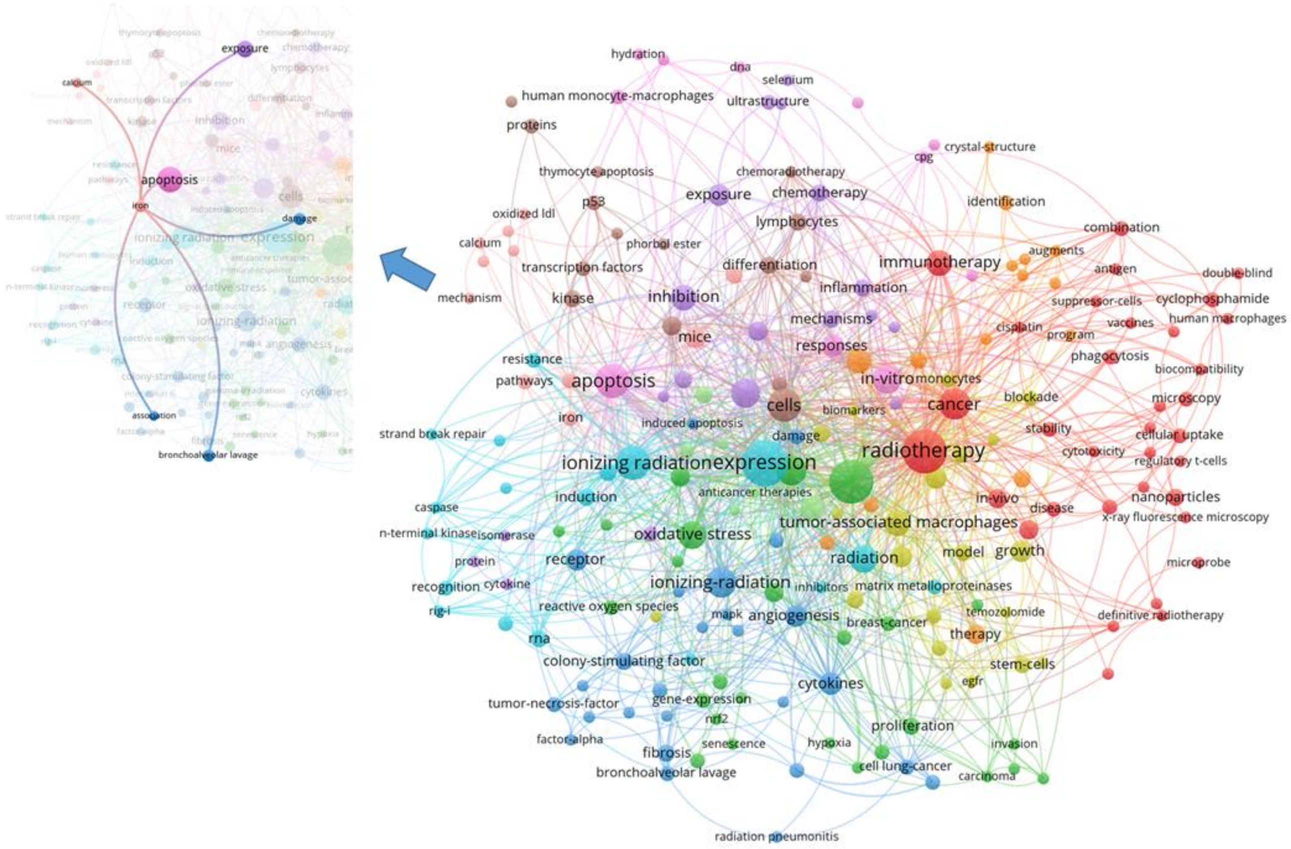


**Figure S4** - Term co-occurrence network of terms associated with macrophage and radiation or X-rays or radiotherapy, evidencing an association between apoptosis and iron. Terms are represented by a node, the size of which is proportional to the frequency of occurrence of the term. The lines indicate that the terms appear together. The terms are divided into clusters represented by different colors

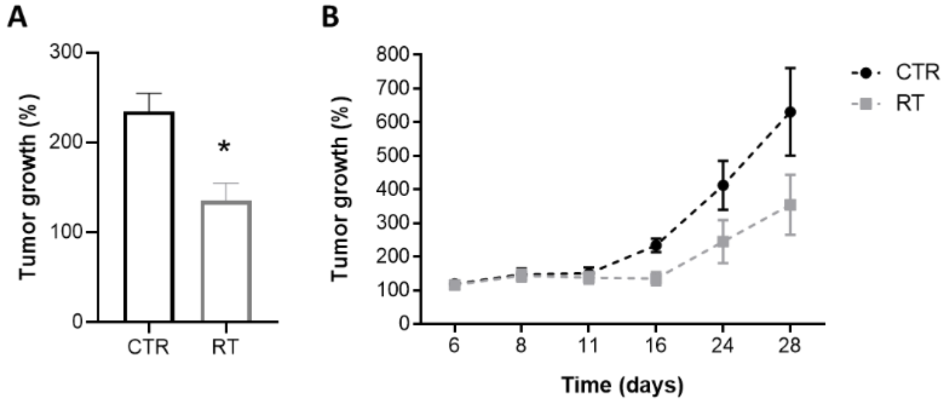


**Figure S5** – Radiotherapy (2 × 5 Gy) significantly reduced relative tumor growth in 4T1 mouse breast tumor model. The relative (%) tumor growth was calculated based on the normalization of tumor volume ($mm^3$) at certain time-point to the initial tumor volume for each animal. Graphs depict tumor growth in irradiated animals (RT) (A) at 6 days after irradiation (d16 post-4T1 injection) and (B) tumor progression 4T1 injection until the end of the experiment, when compared with non-irradiated animals (CTR). Data show the mean ± SEM. Statistical analysis was performed using two-way ANOVA, Sidak´s multiple comparison test, according to [37].

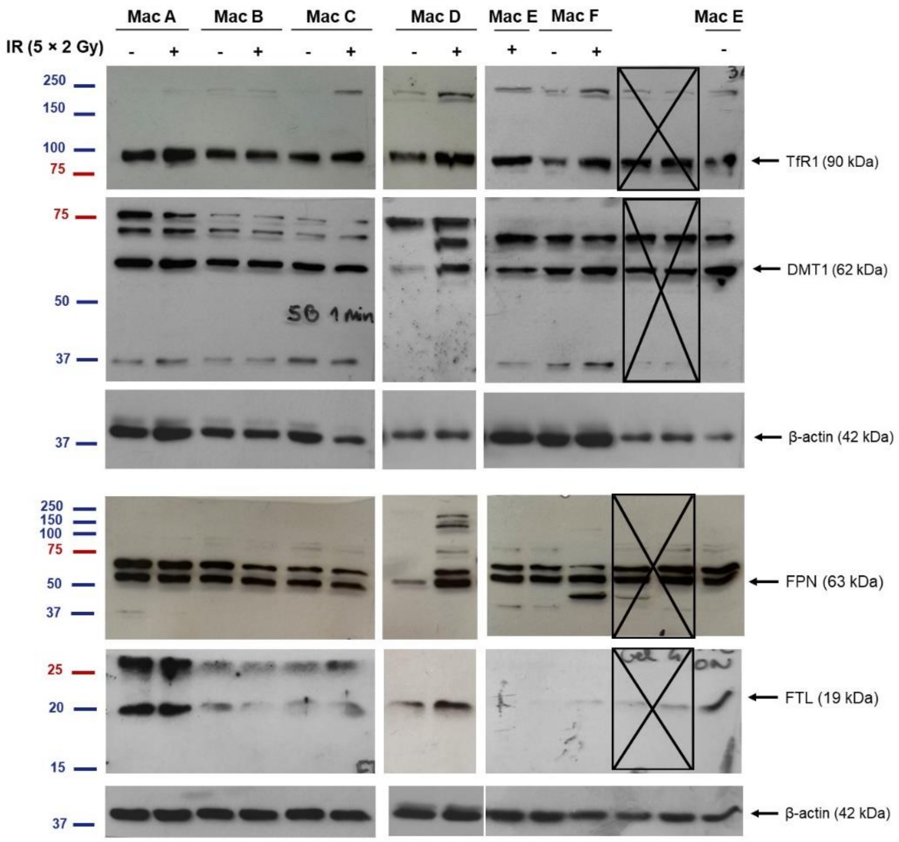
Mac A
Mac B
Mac C
Mac D
Mac E
Mac F
Mac E
IR (5 × 2 Gy)
- + - + - + - + + - + -
250
150
100
75
TfR1 (90 kDa)
75
50
37
DMT1 (62 kDa)
37
β-actin (42 kDa)
250
150
100
75
50
37
FPN (63 kDa)
25
20
15
FTL (19 kDa)
37
β-actin (42 kDa)

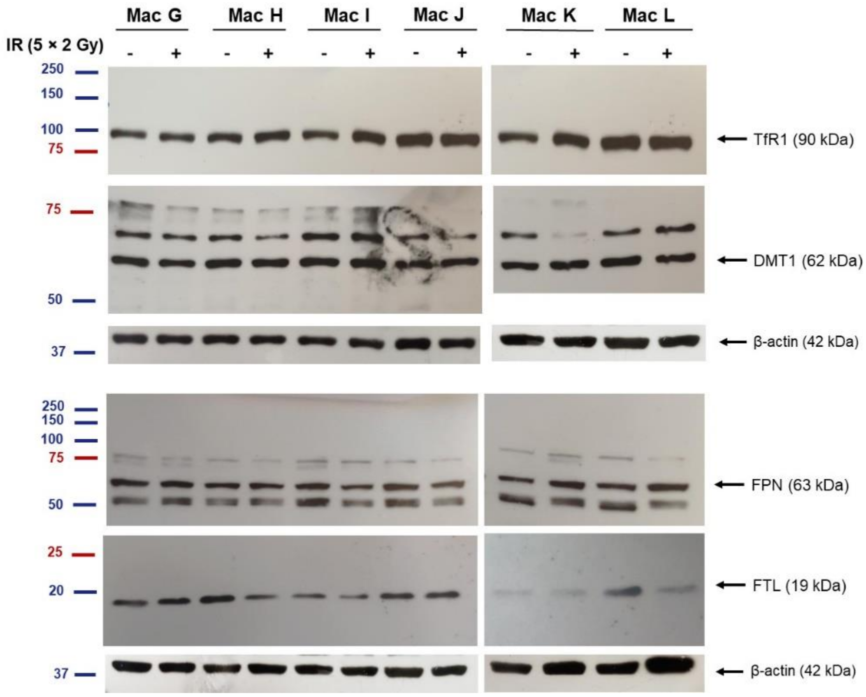
Mac G
Mac H
Mac I
Mac J
Mac K
Mac L
IR (5 × 2 Gy)
- + - + - + - + - + - +
250
150
100
75
TfR1 (90 kDa)
75
50
DMT1 (62 kDa)
37
β-actin (42 kDa)
250
150
100
75
50
FPN (63 kDa)
25
20
FTL (19 kDa)
37
β-actin (42 kDa)

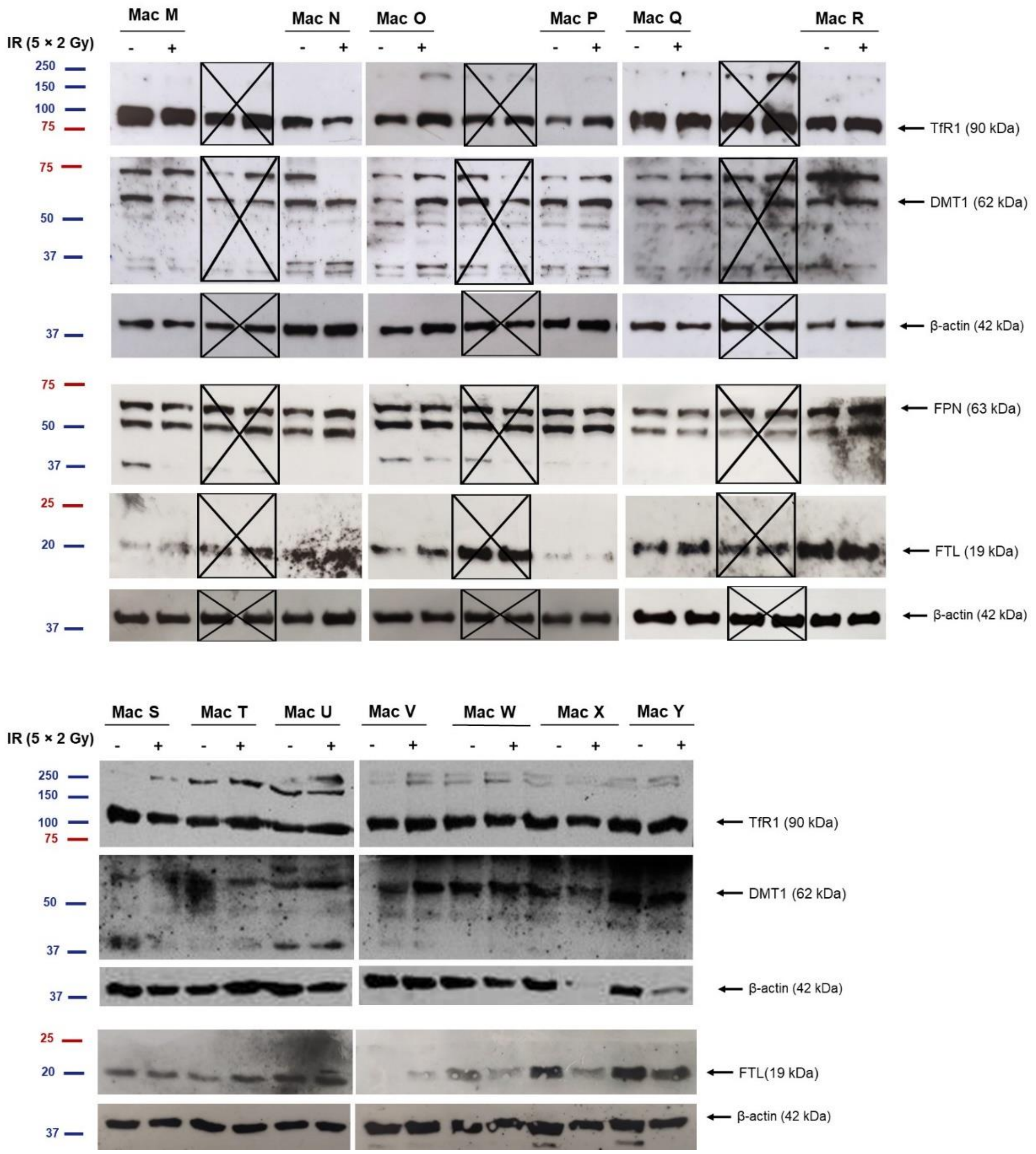


**Figure S6** – Chemiluminescent/scanned images derived from immunoblotting of macrophages whole-cell lysates for TfR1, DMT1, FPN and FTL after exposure to 5 × 2 Gy IR doses. Data were obtained from 5 independent irradiation experiments (n= 17-24 macrophage donors, Mac A-Y). Loading control: β-actin.
